# Bayesian Mechanics of Synaptic Learning Under the Free-Energy Principle

**DOI:** 10.3390/e26110984

**Published:** 2024-11-16

**Authors:** Chang Sub Kim

**Affiliations:** Department of Physics, Chonnam National University, Gwangju 61186, Republic of Korea; cskim@jnu.ac.kr

**Keywords:** free-energy principle, synaptic learning, Bayesian mechanics, continuous-state formulation

## Abstract

The brain is a biological system comprising nerve cells and orchestrates its embodied agent’s perception, behavior, and learning in dynamic environments. The free-energy principle (FEP) advocated by Karl Friston explicates the local, recurrent, and self-supervised cognitive dynamics of the brain’s higher-order functions. In this study, we continue to refine the FEP through a physics-guided formulation; specifically, we apply our theory to synaptic learning by considering it an inference problem under the FEP and derive the governing equations, called Bayesian mechanics. Our study uncovers how the brain infers weight changes and postsynaptic activity, conditioned on the presynaptic input, by deploying generative models of the likelihood and prior belief. Consequently, we exemplify the synaptic efficacy in the brain with a simple model; in particular, we illustrate that the brain organizes an optimal trajectory in neural phase space during synaptic learning in continuous time, which variationally minimizes synaptic surprisal.

## 1. Introduction

The brain’s perception, body movement, and learning are conjointly organized to ensure the homeostasis and adaptive fitness of the embodied agent in the environment. It is tempting to imagine a neural observer in the brain presiding over the cognitive control of higher animals. Such a homunculus idea is untenable and must be discarded in light of the present-day brain theory [1]. However, there is still much distance to a complete scientific understanding of the higher-order functions emerging from brain matter; it demands a comprehension of the profound interplay between the standpoints of scientific reductionism and teleological holism [2,3].

The brain-inspired FEP is a purposive theory that bridges the gap between top-down teleology and bottom-up scientific constructionism. According to the FEP [4,5], all living systems are self-organized to tend to avoid an atypical niche in the environment for existence. The FEP adopts the autopoietic hypothesis [6] and scientifically formalizes the abductive rationale that organisms make optimal predictions and behaviors from uncertain sensory data. To be precise, the FEP suggests an information-theoretic *variational measure* of environmental atypicality, termed *free energy* (FE). The FE objective is technically defined as a functional of the probabilistic generative density specifying the brain’s internal model of sensory data generation and environmental change and an online auxiliary density actuating variational inference in the following sense: the Bayesian brain computes the posterior of the environmental causes of sensory data by minimizing FE, whose detailed continuous-state description can be found in [7]. For discrete-state models of the FEP with discrete time, we recommend [8,9] to readers. FE minimization can also be read as self-demonstrating [10]: this follows from the fact that—as we will see below—FE furnishes a bound on the log marginal likelihood of sensory inputs, where the marginal likelihood can be read as Bayesian model evidence for a generative density or model.

When a Gaussian probability is employed for the variational density [11], the FE becomes a $L_{2}$ norm specified by the Gaussian means and variances, termed the Laplace-encoded FE [7]. Thus, the Laplace-encoded FE provides a scientific base of the $L_{2}$ objectives in a principled manner, which are widely used in machine learning and artificial intelligence. For instance, the optimization function in the predictive-coding framework is proposed to be a sum of the squared (precision-weighted) prediction errors [12]. In addition, the loss function of a typical artificial neural network (ANN) is often written as a sum of squared differences between the ground truth and the predictive entries from the network [13]. Furthermore, it is argued that Gaussian sufficient statistics are encoded by biophysical brain variables, which form the brain’s low-dimensional representations of environmental states. This way, the brain acquires access to the encoded FE for minimization as it becomes fully specified in terms of the brain’s internal states.

Our research over the years has been devoted to developing a continuous-state implementation of FE minimization in a manner guided by physical laws and principles [14,15,16]. We have endeavored to advance the FEP to the point where it coalesces into a unified principle of top-down architecture and material base. Moreover, to promote the FEP to nonstationary problems, we incorporated the fact that the physical brain is in a nonequilibrium (NEQ) steady state and is generally continually aroused by nonstationary sensory stimuli. The functional brain must perform the variational Bayesian inversion of nonstationary sensory data to compute the posterior mentioned above. Previously, we accounted for the brain’s behaviors of perception and motor control, as described by attractor dynamics, and termed the governing equations *Bayesian mechanics* (BM). The BM coordinates the brain’s sensory estimation and motor prediction in neural phase space. In this paper, we make further progress by incorporating the brain’s *synaptic learning* into the BM, which we did not accommodate in our earlier studies. Learning constitutes the crucial brain function of consolidating memory (e.g., via Hebbian plasticity) [17].

In applications of the FEP to generative models, one usually makes a distinction between inference and learning, namely inferring time-dependent states and time-independent parameters, respectively. In this paper, we treat learning as inference by assuming connection weights (i.e., model parameters) are time-dependent, therefore equipping them with dynamics that are coupled to state dynamics (i.e., changes in synaptic weights are coupled to changes in synaptic activity and vice versa). Considering this view, we are concerned with optimizing fluctuations in synaptic plasticity as opposed to long-term potentiation or memory (in which changes in synaptic weights develop very slowly in relation to synaptic activity).

This study aims to provide a simple but insightful formulation of synaptic learning in the Bayesian brain. Our agendas are that the functional brain operates continuously using continuous environmental representations and that synaptic learning is a cognitive phenomenon that may very well be understood when guided by statistical-physical laws. The notion of *cognition* throughout this study is meant to describe the brain’s higher-order capability that involves a top-down, internal model. We consider the NEQ brain a problem-solving matter that cognitively interacts with the environment. To quantify synaptic cognition, we specify the generative densities furnishing the Laplace-encoded FE in a manner to meet the NEQ stationarity and present an FE minimization scheme by practicing the principle of least action (Hamilton’s principle) [18]. The novel contributions derived in this study are discussed in Section 7.

In summary, the current work shows how one can formulate variational (Bayesian) inference in the brain—under the free-energy principle (FEP)—in terms of classical paths of least action. This provides a formal link between the functionalist interpretation of FE minimization as inference and learning and the biophysical implementation that can be described with noisy neuronal dynamics and the ensuing paths of least action. For clarity, we focus on a simple inference problem, namely the postsynaptic response to a presynaptic input. This minimal setup allows us to consider both the postsynaptic neuronal activity and the synaptic weight or efficacy of a generic neuron as encoding the sufficient statistics of (Bayesian) beliefs about the causes of sensory or presynaptic input. This example highlights the intimate relationship between synaptic activity and plasticity in subtending inference and learning, respectively. By dealing with continuous-state spaces and Gaussian random fluctuations, we effectively recover generalized predictive coding by applying Hamilton’s principle of least action to noisy synaptic dynamics. Furthermore, the ensuing BM treatment can accommodate fast dynamics, therefore eschewing gradient-descent schemes in terms of generalized coordinates of motion and providing the basis for a direct link with NEQ thermodynamics.

The remainder of the paper is organized as follows: In Section 2, the single-synapse structure of interest is described. The essence of the FEP is recapitulated, with revision made for synaptic learning, in Section 3. In Section 4, an NEQ formulation is presented, which determines the likelihood and prior densities in the physical brain. Next, in Section 5, the FE objective is identified as a classical action, and the governing equations of synaptic dynamics are derived by exercising the Hamilton principle. The utility of our theory is demonstrated in Section 6, using a simple model. After the discussion in Section 7, a conclusion is given in Section 8.

## 2. Single-Synapse Model

This work concerns the brain’s synaptic learning without considering how environmental processes generate stimuli at the sensory interface; as a parsimonious model, we focus on a single synapse within the brain’s internal environment. For instance, in the hippocampus, the postsynaptic action potential in the dentate gyrus is evoked by a presynaptic signal from the entorhinal cortex caused by a neural signal from other brain areas. Accordingly, the synaptic coupling between two pyramidal neurons in the hippocampus constitutes a single synaptic assembly of interest.

**Figure 1:**
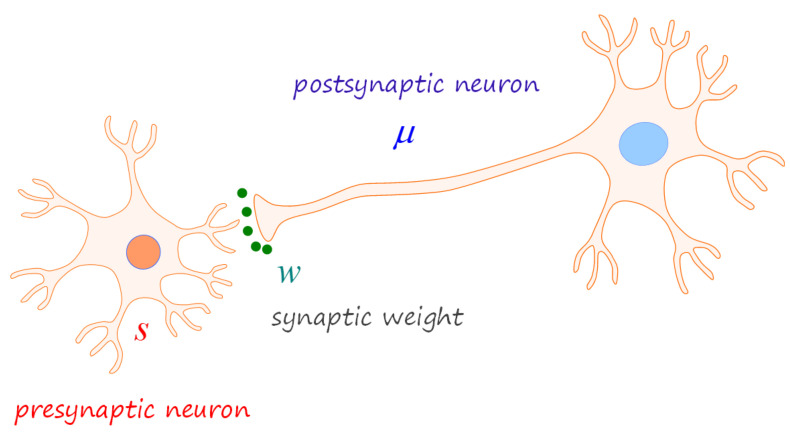
Single synaptic assembly. The postsynaptic neural state μ is neurophysically evoked by the presynaptic signal *s*, mediated by the weight change Δw according to Hebb’s rule ∝sμ. We adopt the Bayesian-inference perspective, suggesting that the brain state μ infers the cause of the presynaptic input *s*, and the weight state *w* makes up the synaptic input-output interface.

We depict the single synaptic model in Figure 1, where the presynaptic and postsynaptic signals are denoted by *s* and μ, respectively; both are the brain’s representations of noisy neural signals. In addition, the synaptic plasticity is mediated by the weight strength denoted by *w*. The considered synaptic structure is generic for all neurons; accordingly, the ensuing formulation applies to other regions of the brain. Please note that we will handle the weight variable *w* as a neurophysical degree of freedom, such as *s* and μ; this approach contrasts with ANN models, where the weights are treated as static parameters.

## 3. Free-Energy Principle for Synaptic Learning

The brain-inspired FEP is built on three hypotheses: (1) The surprisal hypothesis, (2) representation hypothesis, and (3) computability hypothesis. These hypotheses are recapitulated here, along with the revision applied to the synaptic learning problem.

### 3.1. Surprisal Hypothesis

We assume that the presynaptic signals *s* streaming into the synaptic interface are prescribed and focus on the resulting synaptic dynamics. For convenience, we introduce the notation $\tilde{\vartheta }$, with which we collectively denote the postsynaptic variable M and the weight variable W:

$$\tilde{\vartheta }=\left\{M,W\right\}.$$

The variables M and W are random variables that are hidden from the perspective of the postsynaptic neuron and can be regarded as the latent causes of presynaptic input.

The brain’s cognitive goal is to compute the posterior $p(\tilde{\vartheta }|s)$, which the FEP fulfills via variational Bayes in the following manner: First, we define the information-theoretic measure called the Kullback–Leibler (KL) divergence:

(1) $$D_{KL}\left(q\left(\tilde{\vartheta }\right)∥p\left(\tilde{\vartheta }|s\right)\right)=\int d\tilde{\vartheta }q\left(\tilde{\vartheta }\right)\ln\frac{q(\tilde{\vartheta })}{p(\tilde{\vartheta }|s)},$$

which is always positive [19], where $d\tilde{\vartheta }$ denotes dMdW. *Some terminologies*: $q(\tilde{\vartheta })$ is called R-density, which approximates the true posterior $p(\tilde{\vartheta }|s)$ in the variational scheme. The posterior makes up the so-called G-density $p\left(\tilde{\vartheta },s\right)=p\left(\tilde{\vartheta }|s\right)p\left(s\right)$, together with the marginal density p(s) [7]. Second, using the preceding product rule, the above KL divergence can be decomposed to

$$D_{KL}\left(q\left(\tilde{\vartheta }\right)∥p\left(\tilde{\vartheta }|s\right)\right)=F\left[q\left(\tilde{\vartheta }\right),p\left(\tilde{\vartheta },s\right)\right]+\ln p\left(s\right).$$

The functional F on the right-hand side (RHS), which is identified to be

(2) $$F\left[q\left(\tilde{\vartheta }\right),p\left(\tilde{\vartheta },s\right)\right]\equiv \int d\tilde{\vartheta }q\left(\tilde{\vartheta }\right)\ln\frac{q(\vartheta )}{p(\tilde{\vartheta },s)},$$

is the *informational* free energy (FE). Third, the positivity of $D_{KL}$ leads to the inequality

(3) $$-\ln p\left(s\right)\leq F[q\left(\tilde{\vartheta }\right),p\left(\tilde{\vartheta },s\right)].$$

Equation (3) is the mathematical statement of the brain-inspired FEP accounting for biological and cognitive phenomena in a universal manner, which comprises the *surprisal hypothesis*. In the present context, the preceding inequality relates that synaptic learning corresponds to the brain’s minimization of F, which is a proxy for *synaptic surprisal*, −lnp(s), as an upper bound. In practice, it is intractable to determine the marginal density p(s), which provides *synaptic evidence* to the brain. Note here that F is called FE by mimicking thermodynamic FE in physics, which monotonically decreases upon spontaneous isothermal changes in a macroscopic open system. Decreasing the FE of a system open to a reservoir is an alternative statement of the second law of thermodynamics, reconciling with the increase in total entropy of the combined system and reservoir [16].

### 3.2. Representation Hypothesis

According to the inequality [Equation (3)], the brain variationally minimizes F by means of the R-density $q(\tilde{\vartheta })$: when the synaptic interface is elicited by the presynaptic stream *s*, the brain launches $q(\tilde{\vartheta })$, an online approximation of the posterior. In the face of the synaptic stream, the R-density probabilistically represents the uncertain, hidden causes $\tilde{\vartheta }=\left\{M,W\right\}$, which should be matched best with the posterior. Here, we adopt the Laplace approximation for the R-density, which assumes a Gaussian form [20]:

(4) $$q\left(\tilde{\vartheta }\right)=\frac{1}{\sqrt{2\pi {\tilde{\sigma }}^{2}}}\exp\left[-\frac{1}{2{\tilde{\sigma }}^{2}}{\left(\tilde{\vartheta }-\tilde{\mu }\right)}^{2}\right],$$

where $\tilde{\mu }$ and $\tilde{\sigma }$ are the sufficient statistics of the Gaussian density. In particular, we consider that the means denoted by

$$\tilde{\mu }=\left\{\mu ,w\right\}$$

are the coarse-grained representations of high-dimensional $\tilde{\vartheta }=\left\{M,W\right\}$; these are the latent brain variables in low-dimensional neural space [21]. Furthermore, the dependence on the variances $\tilde{\sigma }$ can be eliminated by additional manipulation as elaborated in [7]. Then, under the Laplace approximation, the FE functional reduces to

$$F\left[q\left(\tilde{\vartheta }\right),p\left(\tilde{\vartheta },s\right)\right]=-\ln p\left(\tilde{\mu },s\right)+\mathrm{constants}.$$

The nontrivial part in the reduced expression is the Laplace-encoded FE denoted by *F*:

(5) F(μ,w;s)=−lnp(μ,w,s),

which is a function of only the brain variables μ and *w*, given the presynaptic input *s*. In the present work, the presynaptic input *s* is not a dynamical variable but, instead, is handled as an external time-dependent input.

Here, the brain is assumed to be endowed with the generative density p(μ,w,s) encoded over the evolutionary and developmental time scales. The FEP inequality given in Equation (3) now becomes

(6) −lnp(s)≤F(μ,w;s);

the brain has access to F(μ,w;s) by means of its internal variables μ and *w*. The preceding expression comprises the *representation hypothesis* in the FEP; the brain uses the coarse-grained representations μ and *w* in variationally minimizing synaptic surprisal. Then, by applying the product rule p(μ,w,s)=p(w|μ,s)p(μ,s), the Laplace-encoded FE is completed as

(7) F(μ,w;s)=−lnp(w|μ,s)p(μ,s),

where p(w|μ,s) is the likelihood of the weight strength *w* given a postsynaptic signal μ, and p(μ,s) is the prior regarding the postsynaptic dynamics, which are both subject to the presynaptic input *s*.

Equation (7) is the objective function for synaptic learning under the FEP, furnished with only brain variables, which makes the brain-inspired FEP a biologically plausible theory. Previously, we suggested that all involved probabilities should be specified as NEQ stationary densities derived from the Fokker–Planck equation [16]. This work takes a different approach to determining the NEQ densities, as described in Section 4.

### 3.3. Computability Hypothesis

The brain is endowed with a mechanism that actuates the FE minimization, which comprises the *computability hypothesis* in the FEP. The conventional continuous-state implementation assumes that the brain employs gradient-descent (GD) methods to carry out FE minimization [5]. The GD schemes update the neural activity μ downhill on the FE landscape, which is generally cast in terms of generalized coordinates of motion of all dynamical orders surpassing the second order, namely acceleration [22,23]. It is argued that generalized motion can effectively incorporate the temporal correlation of random fluctuations in stochastic dynamics beyond white noise. However, the idea of generalized motion transcends normative Newtonian physics; thus, its theoretical basis has drawn critical attention in the literature [15,24]. For the weight variable *w*, to incorporate its slower change than the neural activity, a different update rule is applied: for instance, instead of the weight (parameters or hyper-parameters), its rate may be updated under the GD scheme [7]. Researchers have recently extended the applicability of FEP-based GD algorithms to robotics and artificial intelligence problems, emphasizing colored-noise modeling [25]. However, it is significant to note that using GD methods is not legitimate when the environmental inputs vary rapidly, such that the FE landscape becomes non-static (see, for further discussion, Section 7). Our formulation aims at the general time-dependent situation and, thus, avoids using a GD scheme; instead, we identify the FE objective to be a classical action in mechanics and exercise Hamilton’s principle for FE minimization, according to the standard theory [18]. The details are given in Section 5, where we derive the governing equations of motion for synaptic inference regarding the canonical physical variables without invoking generalized motion.

## 4. Nonequilibrium Generative Densities

We argued above that the physical brain probabilistically encodes the representations of the internal and external hidden states (Section 3.2). The encoded probabilities constitute the generative densities that furnish the brain with the FE objective for variational Bayesian inference. Therefore, the generative densities must be specified in terms of the biophysical brain variables in an NEQ steady state. Here, we present a stochastic thermodynamic model for the NEQ densities, viewing the brain as a soft material consisting of neural constituents. This perspective brings us closer to understanding the brain’s NEQ states.

### 4.1. Prior for Postsynaptic Activity

For a simple description, we assume that the brain variable μ obeys an overdamped Langevin dynamics on a mesoscopic scale:

(8) $$\frac{d\mu }{dt}=f\left(\mu ,w;s\right)+\xi ,$$

where *f* and ξ on the RHS are the deterministic and random forces, respectively, causing the neural change. The solution to Equation (8) describes a stochastic path or trajectory μ=μ(t) in continuous-state space. Recall that the neural variable μ is the mean of the R-density probabilistically representing causes of presynaptic inputs online, which may be viewed as a mean field. Also, it is evident that the state transition between two arbitrarily close times described by Equation (8) is Markovian. Further assumptions imposed are as follows: (i) the noise ξ is Gaussian with zero mean, rendering 〈ξ〉=0, and (ii) the noise is delta-correlated (i.e., white) through

(9) $$\langle \xi \left(t^{\prime }\right)\xi \left(t\right)\rangle =\sigma_{\mu }^{2}\delta \left(t^{\prime }-t\right),$$

where $\sigma_{\mu }^{2}$ is the noise strength. In a strict sense, the biological brain is in an NEQ stationary state, whose temperature *T* is distinct from the environmental value; however, here, we consider that the brain is locally in equilibrium characterized by its body temperature. We also assume that the noise strength is given, according to the fluctuation-dissipation theorem [26], as

(10) $$\sigma_{\mu }^{2}=2\gamma_{\mu }^{-1}k_{B}T,$$

where $\gamma_{\mu }$ is the frictional coefficient of the brain matter, and $k_{B}$ is the Boltzmann constant.

Under the prescribed assumptions, we build the transition probability along a trajectory μ=μ(t) as time *t* elapses. To proceed with the derivation, we first note a technical subtlety involved in the white noise ξ(t): it is mathematically ill-defined as the variance is divergent [see Equation (9)]. To address this, the Wiener process, defined through ΔW≡ξΔt, is often conceived. This process introduces a form of coarse-graining over a short time interval Δt, effectively bypassing the singularity of the white noise at an instant time. The Wiener process is also Gaussian with zero mean, with the well-defined variance $\langle {\left(\Delta W\right)}^{2}\rangle =\sigma_{\mu }^{2}\Delta t$. However, one must pay the price for the Wiener recipe when the Riemann integral is performed for state functions over a finite time interval. In our derivation, we adopt the Ito convention, which interprets the integral of Equation (8) over the time interval $\Delta t=t_{n+1}-t_{n}$ as

$$\Delta \mu_{n}=f\left(\mu_{n},w_{n};s_{n}\right)\Delta t+\Delta W_{n},$$

where the first term on the RHS was approximated by choosing the value for f(μ) at the initial time $t_{n}$, and the other terms are $\Delta \mu_{n}=\mu_{n+1}-\mu_{n}$ and $\Delta W_{n}=W_{n+1}-W_{n}$. Next, using the Gaussianity of $\Delta W_{n}$, we define the transition probability p(n+1|n) from the Wiener state $W_{n}$ to the next $W_{n+1}$ as [27]

$$p\left(n+1|n\right)≃\exp\left\{-\frac{1}{2\sigma_{\mu }^{2}\Delta t}{\left(\Delta \mu_{n}-f\left(\mu_{n},w_{n};s_{n}\right)\Delta t\right)}^{2}\right\}.$$

Then, the full Markovian transition over N(=t/Δt) time steps during the finite time $0\leq t^{\prime }\leq t$ can be built as

$$\prod_{n=0}^{N-1}p\left(n+1|n\right)≃\exp\left\{-\frac{\Delta t}{2\sigma_{\mu }^{2}}\sum_{n}{\left(\frac{\Delta \mu_{n}}{\Delta t}-f\left(\mu_{n},w_{n};s_{n}\right)\right)}^{2}\right\}.$$

As a final step, we take the continuous limit Δt→0 in the preceding expression and obtain the path probability p(μ,s), up to a normalization constant, as

(11) $$p\left(\mu ,s\right)∼\exp\left\{-\frac{1}{2\sigma_{\mu }^{2}}\int_{0}^{t}dt^{\prime }{\left(\frac{d\mu }{dt^{\prime }}-f\left(\mu ,w;s\right)\right)}^{2}\right\},$$

which is known as the Onsager-Machlup function [28].

The above Onsager–Machlup expression specifies the transition probability of the neural state μ, given initial condition μ(0), along the continuous path μ=μ(t). It represents the prior density p(μ,s) in Equation (7) accounting for the brain’s belief about or acquired knowledge regarding how the postsynaptic activity μ behaves.

### 4.2. Likelihood of Synaptic Change

Neurotransmitter transport at the synaptic interface mediates synaptic coupling between two neurons, which is often effectively described by the weight variable *w*. We assume that the brain is endowed with an internal model of weight dynamics leveraging learning, where learning constitutes the crucial brain function of consolidating memory (e.g., via long-term potentiation).

We consider the synaptic weight *w* a time-dependent variable rather than a static parameter, and the synaptic plasticity is described by its rate $\dot{w}=dw/dt$. We propose that, similar to Equation (8), the synaptic plasticity is governed by the stochastic equation:

(12) $$\frac{dw}{dt}=h\left(w,\mu ;s\right)+\chi ,$$

where *h* is the biophysical force causing the weight change and χ is the additive white noise associated with the synaptic process. The noise is assumed to be Gaussian with zero mean and delta-correlated: $\langle \chi \left(t\right)\chi \left(t^{\prime }\right)\rangle =\sigma_{w}^{2}\delta \left(t-t^{\prime }\right)$, where $\sigma_{w}^{2}$ being the noise strength.

Next, to smooth the temporal singularity associated with the white noise χ, we consider the Wiener process $\Delta W_{\chi }=\chi \Delta t$, which is also Gaussian about zero mean with the well-defined variance, $\langle {\left(\Delta W_{\chi }\right)}^{2}\rangle =\sigma_{w}^{2}\Delta t$. Then, we proceed with the same formulation as in Section 4.1 to specify the NEQ likelihood density p(w|μ,s). The result is given as

(13) $$p\left(w|\mu ,s\right)∼\exp\left\{-\frac{1}{2\sigma_{w}^{2}}\int_{0}^{t}dt^{\prime }{\left(\frac{dw}{dt^{\prime }}-h\left(w,\mu ;s\right)\right)}^{2}\right\},$$

which represents the Onsager–Machlup transition probability along the continuous path w=w(t), subject to the initial condition w(0).

In obtaining the above prior and likelihood densities—namely Equations (11) and (13), respectively—we assumed that the random fluctuations in the neuronal dynamics were delta-correlated (i.e., white noises). The brain signals, in contrast, evidently reveal the frequency spectrum reflecting color-correlated dynamics [29], which supports the criticality idea in the brain [30]. In this work, we consider only the ideal white noise for the sake of practical illustration of determining the NEQ brain densities in a physics-grounded manner. Obtaining an analytic expression for the NEQ densities is intractable under general conditions, even in the steady state [16]; they are usually assumed to be an instant Gaussian set by the Gaussian random noises imposed on the Langevin description [4,5].

## 5. Bayesian Mechanics: Computability of Synaptic Learning

The synaptic FE landscape is generally non-static, as the presynaptic input *s* in the generative densities is explicitly time-dependent. Thus, it is anticipated that the usual GD implementation on the FE landscape will fail. Accordingly, we formulate the brain’s computability under nonstationary conditions, facilitating nonautonomous neural computation.

Here, we substitute the Onsager–Maclup path probabilities [Equations (11) and (13)] into Equation (7), and obtain the mathematical expression for Laplace-encoded synaptic FE as follows:

(14) $$F=\int_{0}^{t}L\left(\mu ,w;\dot{\mu },\dot{w};s\right)dt^{\prime },$$

where the integrand L is expressed as

(15) $$L\left(\mu ,w;\dot{\mu },\dot{w};s\right)\equiv \frac{1}{2\sigma_{\mu }^{2}}{\left(\frac{d\mu }{dt^{\prime }}-f\left(\mu ,w;s\left(t^{\prime }\right)\right)\right)}^{2}+\frac{1}{2\sigma_{w}^{2}}{\left(\frac{dw}{dt^{\prime }}-h\left(\mu ,w,s\left(t^{\prime }\right)\right)\right)}^{2}.$$

Please note that in Equation (15), we concretely displayed the autonomous dependence on the variables μ and *w* and the nonautonomous dependence (explicit time-dependence) on the input *s* through the generative functions *f* and *h*.

Equation (14) manifests a specific association of the FE objective *F* with the mathematical object L; namely *F* is given as a time-integral of L. This observation is reminiscent of the relation between the action and Lagrangian in classical mechanics [18]. Accordingly, by analogy, if we identify *F* as an effective *action* S and the integrand L as an effective *Lagrangian* for the brain’s cognitive computation, the FE minimization—which is mathematically performed by δF=0 under the FEP—is precisely mapped to exercising Hamilton’s principle, δS=0. Then, the Euler-Lagrange equations of motion for determining the optimal trajectories μ(t) and w(t) will follow in a straightforward manner, constituting the synaptic BM. Note the temperature dependence of the Lagrangian [Equation (15)] via the noisy strengths $\sigma_{\mu }^{2}$ and $\sigma_{w}^{2}$—Equation (10)—which makes L a *thermal* Lagrangian [28]. See also [31] for a path integral formulation in generalized coordinates of motion.

Here, working in the Hamiltonian description is more suitable for our purposes. To this end, we carried out a Legendre transformation of L to derive an effective *Hamiltonian* H:

$$H=\dot{\mu }\frac{\partial L}{\partial \dot{\mu }}+\dot{w}\frac{\partial L}{\partial \dot{w}}-L.$$

The outcome is expressed as

(16) $$H=\frac{p_{\mu }^{2}}{2m_{\mu }}+\frac{p_{w}^{2}}{2m_{w}}+p_{\mu }f\left(\mu ,w;s\right)+p_{w}h\left(w,\mu ;s\right).$$

In the preceding expression of Hamiltonian, the new variables $p_{\mu }$ and $p_{w}$ appear, which are mechanically conjugate to the variables μ and *w*, respectively. They are determined from the definitions:

(17) $$p_{\mu }=\frac{\partial L}{\partial \dot{\mu }}\;\mathrm{and}\;p_{w}=\frac{\partial L}{\partial \dot{w}}.$$

Additionally, the constants $m_{\mu }$ and $m_{w}$ were defined as

(18) $$m_{\mu }=1/\sigma_{\mu }^{2}\;\mathrm{and}\;m_{w}=1/\sigma_{w}^{2},$$

which are measures of the respective precisions of the probabilistic generative models, Equations (11) and (13). Equation (10) suggests that the generative precisions are a biophysical constant specified by the body temperature and the friction of the brain matter. A few points about the Hamiltonian H are noteworthy: The variables (μ, *w*) and ($p_{\mu }$, $p_{w}$) correspond to *positions* and *momenta*, respectively, and the generative precisions $m_{\mu }$ and $m_{w}$ may be interpreted as a *neural mass* as a metaphor. The Hamiltonian is not breakable into the kinetic and potential energies, as the third and fourth terms on the RHS in Equation (16) are given as a product of momentum and position variables. The H function does not furnish a conservative energy surface due to its explicit time-dependence through the presynaptic signal s(t).

The generative functions *f* and *h* for synaptic learning were introduced in Equations (8) and (12) without specifying them; they are the biophysical forces driving synaptic dynamics at the neuronal level. We now specify them by the following models:

(19) $$\begin{array}{ccc} f(\mu ,w;s) & = & -\gamma_{\mu }\left(\mu -\mu_{d}\right)+ws, \end{array}$$

(20) $$\begin{array}{ccc} h(w,\mu ;s) & = & -\gamma_{w}\left(w-w_{d}\right)+s\mu . \end{array}$$

The first terms on the RHSs, involving the damping coefficients $\gamma_{\mu }$ and $\gamma_{w}$, prevent the unlimited growth of μ and *w* [32]. The linear damping models may be replaced with a nonlinear alternative; for instance, the modified $-\gamma_{w}s^{2}\left(w-w_{d}\right)$ may be used in Equation (20) [33]. The second term ws on the RHS of Equation (19) describes the presynaptic input weighted by *w*. Moreover, the term sμ in Equation (20) accounts for Hebb’s rule; one can explore anti-Hebbian learning by inverting its sign. The extra parameters $\mu_{d}$ and $w_{d}$ are the steady-state values of μ and *w*, respectively, without driving terms ws and sμ. After substituting Equations (19) and (20) into Equation (15) and evaluating Equation (17), one can determine the neural representations of the momenta $p_{\mu }$ and $p_{w}$. The results are given as

(21) $$\begin{array}{cc} & p_{\mu }=m_{\mu }\left(\dot{\mu }-f\right), \end{array}$$

(22) $$\begin{array}{cc} & p_{w}=m_{w}\left(\dot{w}-h\right). \end{array}$$

Please note that momentum represents the discrepancy between the state rate and its prediction from the generative model, which corresponds to (precision-weighted) *prediction error* in predictive-coding theory (see discussion in Section 7).

Having specified the synaptic Hamiltonian given in Equation (16), we now derive Hamilton’s equations of motion by following the standard procedure [18]. We present only the outcome without showing intermediate steps:

(23) $$\begin{array}{ccc} \dot{\mu } & = & \frac{1}{m_{\mu }}p_{\mu }-\gamma_{\mu }\left(\mu -\mu_{d}\right)+ws, \end{array}$$

(24) $$\begin{array}{ccc} \dot{w} & = & \frac{1}{m_{w}}p_{w}-\gamma_{w}\left(w-w_{d}\right)+s\mu , \end{array}$$

(25) $$\begin{array}{ccc} {\dot{p}}_{\mu } & = & \gamma_{\mu }p_{\mu }-sp_{w}, \end{array}$$

(26) $$\begin{array}{ccc} {\dot{p}}_{w} & = & \gamma_{w}p_{w}-sp_{\mu }. \end{array}$$

**Figure 2:**
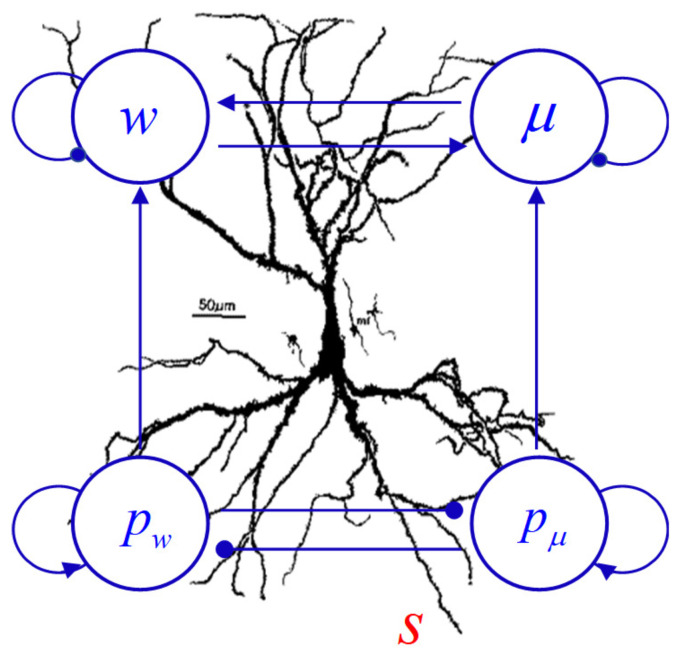
Schematic of the neural circuitry. The diagram manifests the workings of the synaptic BM: the presynaptic input s(t) drives the interconnected, recurrent dynamics among the state (w,μ) and momentum $\left(p_{w},p_{\mu }\right)$ variables. The links depicted by arrowheads indicate excitatory coupling within a neural unit or between two neural units, whereas the dot-head links indicate inhibitory coupling.

The resulting Equations (23)–(26) are a set of coupled differential equations for four dynamical variables μ, *w*, $p_{\mu }$, and $p_{w}$, subject to the time-dependent input source *s*, which constitute the synaptic BM governing *co-evolution* of the state and weight variables. In Figure 2, we show the neural circuitry implied by the derived BM. We argue that the functional behavior depicted in the circuitry is generic in every synapse in the brain, similar to every cortical column in the neocortex behaving as a sensorimotor system performing the same intrinsic function [34].

For a more compact description, we shall define the cognitive state Ψ as a column vector in four-dimensional *phase space*:

$$\Psi^{T}=\left(\mu ,w,p_{\mu },p_{w}\right)\equiv \left(\psi_{1},\psi_{2},\psi_{3},\psi_{4}\right),$$

where *T* denotes a transpose operation. Then, the preceding Equations (23)–(26) can be compactly expressed as

(27) $$\dot{\Psi }=R\Psi +I,$$

where R is a 4×4 matrix identified as

(28) $$R=\left(\begin{array}{cccc} -\gamma_{\mu } & s & 1/m_{\mu } & 0\\ s & -\gamma_{w} & 0 & 1/m_{w}\\ 0 & 0 & \gamma_{\mu } & -s\\ 0 & 0 & -s & \gamma_{w} \end{array}\right),$$

and the inhomogeneous vector I is identified to be

(29) $$I^{T}=\left(\gamma_{\mu }\mu_{d},\gamma_{w}w_{d},0,0\right).$$

Equation (27) can be formally integrated to bring about the solution:

(30) $$\Psi \left(t\right)=e^{\int_{0}^{t}R\left(t^{\prime }\right)dt^{\prime }}\Psi \left(0\right)+\int_{0}^{t}dt^{\prime }e^{\int_{t^{\prime }}^{t}R\left(\tau \right)d\tau }I,$$

where the first term on the RHS is a homogeneous solution, given the initial condition Ψ(0), and the second term is the inhomogeneous solution, driven by the source I. The formal solution represents a continuous path in 4-dimensional phase space, which variationally optimizes the FE objective [Equation (14)]. Please note that the trace of R vanishes identically; that is, Tr(R)=0. Accordingly, the sum of its eigenvalues must equal zero, which we use as a consistency condition in the numerical calculation presented in Section 6. In addition, when the presynaptic signal is constant or saturates in time, the fixed point $\Psi_{eq}$ can be obtained analytically:

(31) $$\Psi_{eq}^{T}=\left(\frac{\mu_{d}+s_{\infty }w_{d}/\gamma_{\mu }}{1-s_{\infty }^{2}/\gamma_{\mu }\gamma_{w}},\frac{w_{d}+s_{\infty }\mu_{d}/\gamma_{w}}{1-s_{\infty }^{2}/\gamma_{\mu }\gamma_{w}},0,0\right),$$

where we used the notation $s_{\infty }=s\left(t\to \infty \right)$.

## 6. Numerical Illustration

To exemplify the workings of the BM conducting synaptic inference, we numerically integrated Equations (23)–(26) using Mathematica 14.1. The Mathematica code we wrote is provided in Appendix A.

### 6.1. Free Parameters

Six free parameters appear in the BM, which need to be fixed for numerical purposes; the values we choose are displayed in Table 1. The neural masses $m_{\mu }$ and $m_{w}$ are a measure of inferential precision, defined to be the inverse noise strengths [Equation (18)]. The frictional coefficients denoted by $\gamma_{\mu }$ and $\gamma_{w}$ appear in the generative functions [Equations (19) and (20)], which we set $\gamma_{\mu }=10\gamma_{w}$ to account for the slower weight dynamics compared to the neuronal activity. Furthermore, the parameters $\mu_{d}$ and $w_{d}$ in the inhomogeneous vector [Equation (29)] represent the brain’s prior belief about the postsynaptic and weight values before the presynaptic input arrives.

### 6.2. Static Presynaptic Input

We first present the numerical outcome when the synapse delineated in Figure 1 is evoked by a static presynaptic signal, which we set as s=5.

**Figure 3:**
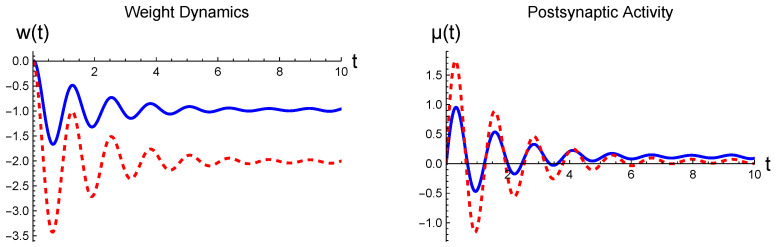
Synaptic dynamics evoked by the static presynaptic input s=5. The parameter values that we used to produce the graphs are displayed in Table 1; the blue and red curves are the results from the upper and lower parameter sets, respectively. The initial conditions were chosen as μ(0)=0 and w(0)=0. All curves are in arbitrary units.

Figure 3 shows the synaptic response of *w* and μ to the prescribed input from two different parameter sets displayed in Table 1. Both cases exhibit transient harmonic behaviors: The figure manifests that, in response to the static input, the magnitude of the output signals initially increases from the starting value 0. Then, they approach the corresponding fixed points in a sinusoidal manner; $\left(w_{eq},\mu_{eq}\right)=\left(-1.0,0.1\right)$ for the solid curve and $\left(w_{eq},\mu_{eq}\right)=\left(-2.0,0.0\right)$ for the dotted curve. The transient harmonic behavior is attributed to imaginary eigenvalues of the matrix *R* for the chosen parameters. The plots for $p_{\mu }$ and $p_{w}$ are not shown because we exploit dynamics near the fixed points, where their values are zero [see Equation (31)]. In general, the full synaptic dynamics undergoes in 4-dimensional phase space.

**Figure 4:**
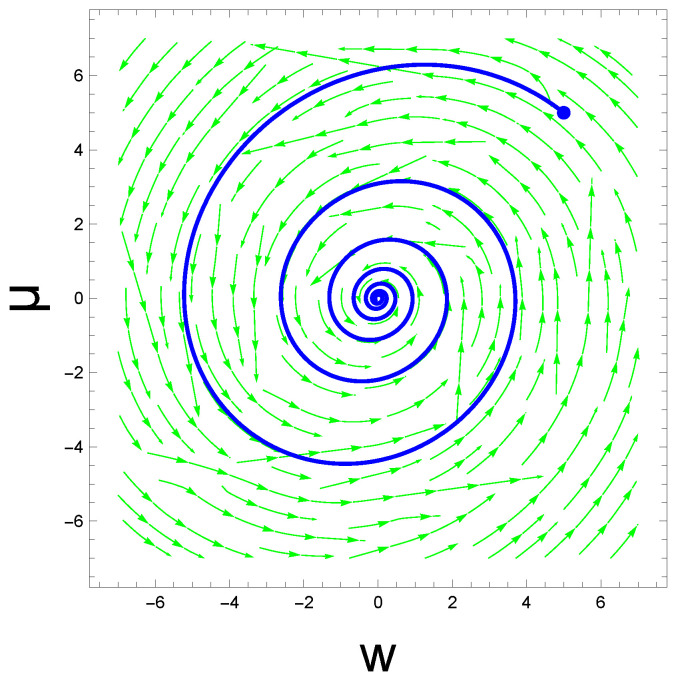
Continuous path driven by the static presynaptic input s=5: The initial condition was chosen at (w,μ)=(5,5), marked by the blue dot. Additionally, for illustrational purposes, we set $\mu_{d}=0$ and $w_{d}=0$, while other parameter values were the same as in Table 1. All curves are in arbitrary units.

In Figure 4, we illustrate a trajectory in the state space spanned by (w,μ); the numerical conditions are described in the caption. One can observe the spiral approach to the fixed point (0,0), starting from the initial condition (5,5), arbitrarily chosen for illustrative purposes. Again, the momentum representations are not drawn, as their values remain near the equilibrium point in the considered linear dynamics. Please note that the irregularity in the background streamlines is due to the noise in the presynaptic input, reflecting the fact that the brain deterministically predicts cognitive outcomes only on average. The illustrated trajectory is critical to understanding the unconscious cognition of weight change and postsynaptic output. The temporal course is conditioned on the presynaptic input in the Bayesian brain—a crucial context for our research.

### 6.3. Nonstationary Presynaptic Inputs

Here, we present the numerical results of when the nonstationary presynaptic inputs drive the BM.

**Figure 5:**
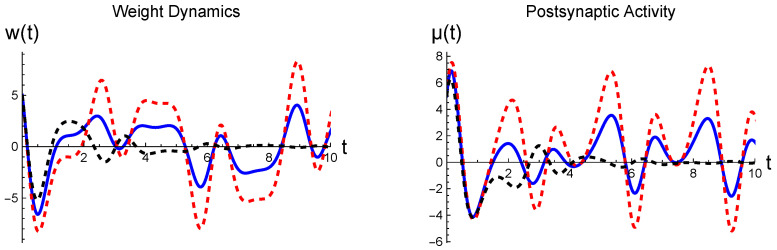
Synaptic dynamics evoked by s(t)=5cost+η, where η represents a random fluctuation: The blue solid and red dotted curves are the results from the parameter values displayed in Table 1; in addition, we include the black dotted curve from $\mu_{d}=0$ and $w_{d}=0$, while other parameter values remain the same. For all data, the initial condition was chosen at (w,μ)=(5,5). All curves are in arbitrary units.

First, in Figure 5, we illustrate the weight dynamics and the postsynaptic activity resulting from the sinusoidally varying presynaptic input. In this case, the continual harmonic driving causes the output signals to retain their oscillatory behavior and not tend to a fixed point. The output signals exhibit both positive and negative portions because we considered the voltage-dependent plasticity, aiming at the continuous change, which could induce the negative voltage response [35]. In contrast, only positive signals would be produced if we considered the spike-timing-dependent plasticity. The momentum variables are not drawn as we follow dynamics near the fixed point in neural phase space, where they remain nearly zero. It also needs to be understood that the weight dynamics are ten times slower than the postsynaptic activity, as we assumed that the postsynaptic signal decays ten times faster (see Table 1). The same interpretation applies to Figure 3.

**Figure 6:**
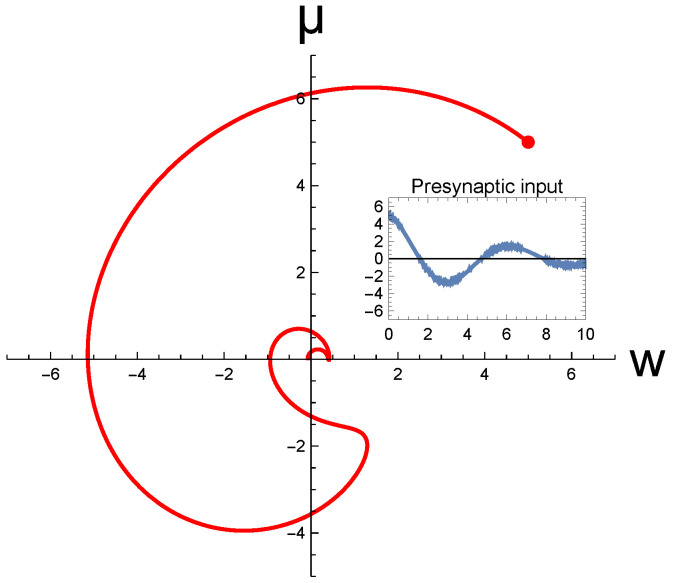
Continuous trajectory in neural state space. The inset shows the transient input signal driving synaptic dynamics, $s\left(t\right)=5e^{-t/5}\cos t+\eta$, where η denotes a noise. The initial values of the weight *w* and postsynaptic signal μ were chosen at (w,μ)=(5,5), marked by a red dot; the neural trajectory manifests a continuous approach to the fixed point (0,0). All curves are in arbitrary units.

Next, in Figure 6, we illustrate the neural trajectory in two-dimensional state space produced by the transient input signal (which is shown as an inset). It demonstrates that the brain’s synaptic computation follows a continuous approach to the fixed point—in this case, the origin—starting from the chosen initial state (w,μ)=(5,5). In numerically integrating the synaptic BM to obtain the trajectory, the parameter values were chosen from Table 1 except that, for illustrational purposes, the values for $\mu_{d}$ and $w_{d}$ were both set to be 0. Please note that we did not draw the streamlines in the figure as the presynaptic input is time-dependent, so the streamlines vary at every moment in the trajectory’s course. In Figure 4, by contrast, the input was static so that we could delineate the streamlines. Notably, our BM theory allowed us to handle the nonautonomous problem induced by the nonstationary presynaptic inputs. On the other hand, the computability of the usual GD minimization for the present problem is questionable as the FE landscape is non-static, as described in Section 3.3.

The neural paths we numerically illustrated in the current Section are optimal trajectories minimizing synaptic surprisal; meanwhile, the actual minimization was performed on the variational FE objective [see Equation (14)] under the FEP [see Equation (6)]. We emphasize that the learned trajectories were self-organized, given the values of material parameters of the brain matter. In essence, the brain’s learning process is unsupervised (in the language of machine learning); this means that the brain does not require any external *label* to guide its learning, demonstrating its self-learning capability.

## 7. Discussion

The idea that the brain is a neural observer (reinforcer or problem-solving matter) is implicit in the brain-inspired FEP, which is capable of perceiving, learning, and acting on the external and internal milieus; this renders the FEP a purposive theory. On the other hand, brain functions must emerge from the brain matter in a manner that obeys physical laws and principles; the neural substrates afford the biological base for the brain’s high-order capability. Thus, it is significant to recognize that the working FE objective is not a single measure but, instead, an architecture hybridizing the teleological rationale and biophysical realities.

In this study, we continued our endeavor for the continuous-state implementation of the promising FEP as a universal biological principle. Specifically, we applied our theory to synaptic learning and exemplified the learning dynamics as an inference problem under the FEP. The noteworthy contributions from our effort are discussed below:

(i) Equation (14) is the FE objective in our theory, which suggests that the FE conducts itself as a classical (cognitive) action in Hamilton’s principle; that is, S=F. We obtained the result by deriving the Onsager-Machlup representations for the NEQ generative densities and inserting them into the Laplace-encoded FE. In our previous studies [14,15,16], in contrast, the cognitive action was identified as a time-integral of the FE, S=∫Fdt, under the ergodic assumption. The ergodicity asserts that the ensemble average of surprisal (i.e., the negative log sensory evidence) equals the corresponding temporal average; however, it is difficult to justify the ergodicity idea in the brain. In the present work, we bypassed the ergodic assumption using the more physics-grounded NEQ densities and avoided employing the generalized coordinates of motion; this grounds FEP computations in the physics of stochastic dynamical systems under NEQ conditions.

(ii) The weight variables change over time due to biophysical factors such as the opening of channels at the synapse, through which neurotransmitters transfer in a complex time-dependent manner. Accordingly, we treated the synaptic weights *w* as a dynamical variable co-evolving with the state variables in completing the synaptic BM. In contrast, the weights are handled as a static parameter in the widely utilized ANNs in machine learning. Furthermore, in the frameworks of ANNs, a nonlinear activation scheme—such as the sigmoidal function or ReLU (rectified linear unit)—rectifies the network output value [13]. Our biophysics-informed treatment does not use engineering manipulation to regulate the outcome; instead, the learning smoothly follows the continuous BM. We add that one may employ different biophysical models from our Langevin dynamics, such as Izhikevich neurons [36] at the neuronal level or neural field models on a mesoscopic scale [37], and apply our framework to derive a desired BM.

(iii) The momentum representations we derived [see Equations (21) and (22)] match with the theoretical construct of prediction error in predictive-coding theory [12,38]. Empirical evidence of error neurons has recently been reported, which encodes prediction errors in the mouse auditory cortex [39]. Such a finding provides a neural basis for our theory. However, the differentiation between predictive and error units within a cortical column is still controversial, mainly because of insufficient electrophysiological recordings. Although there is no concrete agreement, the compartmental neuron hypothesis seems to suit the neuronal scenario of functional distinguishability [40,41], which argues that pyramidal neurons in the cortex are functionally organized such that feedback and feed-forward signals are sent to outer layers (L1) and middle layers (L5), respectively. In this case, our state representations correspond to feedback channels via apical dendrites and momentum representations to feed-forward channels via basal dendrites in somas. The Hebbian sign of Equation (20) can be either positive or negative, and one can implement spiking predictive coding with the former and the dendrite predictive coding with the latter.

(iv) Data learning via ANNs has become a formidable scientific tool [42], and much attention is drawn to theoretical questions on how and why they work [43]. This paper suggested that the brain-inspired FEP underlies the widely used $L_{2}$ objective in machine learning algorithms. The $L_{2}$ minimization is implemented using a GD with respect to the weights connecting layers, rendering back-propagation of the input-output error reduction in a feed-forward architecture. However, strictly speaking, the validity of GD updating is limited to situations when the inputs are static or quasi-static. For continuous nonstationary inputs such as a video stream, a bidirectional recurrent NN (RNN) is employed [44]; the RNN sends converted time-series inputs to a pre-structured deep network and performs GD by incorporating a feedback loop to predict the sequential outputs. Our BM formulation, in contrast, handles nonstationary learning in a genuinely continuous manner, offering a fresh perspective. The brain integrates the BM to learn a continuous optimal trajectory in neural phase space by minimizing the FE objective rather than producing a sequential output. We hope that our physics-guided approach will provide further useful insights into the practice of ANN methodologies in continuous time.

## 8. Conclusions

In the continuous-state FEP framework, we framed synaptic learning in the context of minimizing the FE objective, the upper bound for synaptic surprisal in the brain, and we derived the BM by implementing FE minimization in a physics-guided manner. Consequently, we revealed that the brain conducts synaptic learning by integrating the BM to find an optimal trajectory in the reduced-dimensional neural phase space.

## Figures and Tables

**Table 1:** Parameter values used to produce the data.

	$m_{\mu }$	$m_{w}$	$\gamma_{\mu }$	$\gamma_{w}$	$\mu_{d}$	$w_{d}$
Solid	5	0.5	1	0.1	5	5
Dotted	5	0.5	1	0.1	10	0

## Data Availability

We provide the computer code in Appendix A, which we wrote to generate the data using Mathematica 14.1.

## Abbreviations

The following abbreviations are used in this manuscript:

FE	free energy
FEP	free-energy principle
BM	Bayesian mechanics
KL	Kullback–Leibler
GD	gradient descent
NEQ	nonequilibrium
ANN	artificial neural network

## Appendix A. Mathematica Code

This appendix contains the Mathematica code that integrates the Bayesian mechanics of synaptic learning, which is schematized in the neural circuitry depicted in Figure 2.

ClearAll["Global’*"]

** **

(* Presynaptic signals to choose *)

seq = 5;

znoise := RandomReal[-.02, .02];

(* s[t** **] := seq + znoise;*) (* Noisy static presynaptic input *)

(* s[t** **]:= seq*Cos[t]+znoise;*) (* Oscillatory presynaptic input *)

s[t** **]:= seq*Exp[-t/5]*Cos[t]+znoise; (* Transient oscillatory presynaptic input *)

Plot[s[t], {t, 0, tmax},AxesLabel -> {Style["t", FontSize -> 15],Style["s(t)", FontSize -> 15]}, PlotRange -> All];

(* Setting up the values of parameters in generative functions: g, f, and h *)

mmu = 5; mw = 0.5;

mud = 0; wd = 0;

gamma1 = 1.0; gamma2 = 0.1;

sign = -1 (* The Hebbian sign *);

tn = 1.3;

tdomain = tmax/tn;

** **

(* We use NDSolve to numerically integrate the BM *)

{musol, wsol, pmusol, pwsol} = NDSolveValue[{mu’[t] == pmu[t]/mmu - gamma1*(mu[t] - mud) + s[t]*w[t], w’[t] == pw[t]/mw - gamma2*(w[t] - wd) + sign*s[t]*mu[t], pmu’[t] == gamma1*pmu[t] - sign*s[t]*pw[t], pw’[t] == gamma2*pw[t] - s[t]*pmu[t], mu[0] == 5, w[0] == 5, pmu[0] == -0.0001, pw[0] == 0.0001}, { mu, w, pmu, pw}, {t, 0, tdomain}]

fxpt1 = {mufix, wfix} = {musol[10], wsol[10]} // N;

grmu1 = Plot[musol[t], {t, 0, tdomain}, PlotRange -> {{0, 10}, All}];

grw1 = Plot[wsol[t], {t, 0, tdomain}, PlotRange -> {{0, 10}, All}];

grpmu1 = Plot[pmusol[t], {t, 0, tdomain}, PlotRange -> {{0, 10}, {-.1, .1}}];

grpw1 = Plot[pwsol[t], {t, 0, tdomain}, PlotRange -> {{0, 10}, All}];

** **

(* Draw graphs *)

GraphicsGrid[{{grw1, grmu1}}] (* This produces the blue curves in Figure 5. *)

ParametricPlot[{wsol[t], musol[t]}, {t, 0, tdomain}, PlotRange -> Full] (* This produces the red trajectory in Figure 6. *)

** **

(* One can easily modify the above code to generate other graphs in the paper. *)

(* One needs to monitor the outcome because the numerical integration blows up for times later than tdomain. *)
